# Sitting time in Germany: an analysis of socio-demographic and environmental correlates

**DOI:** 10.1186/1471-2458-13-196

**Published:** 2013-03-06

**Authors:** Birgit Wallmann-Sperlich, Jens Bucksch, Sylvia Hansen, Peter Schantz, Ingo Froboese

**Affiliations:** 1 Institute of Health Promotion and Clinical Movement Science, German Sports University, Köln, D-50933, Germany; 2 Centre for Health, German Sports University Cologne, Köln, D-50933, Germany; 3 WHO Collaborating Centre for Child and Adolescent Health Promotion, School of Public Health, Bielefeld University, Bielefeld, D-33615, Germany; 4 Faculty of Health, Medicine and Life Sciences, Maastricht University, Maastricht, 6229 ER, The Netherlands; 5 The Research Unit for Movement, Health and Environment, The Åstrand Laboratory, GIH – The Swedish School of Sport and Health Sciences, Stockholm, SE-114 86, Sweden; 6 Department of Health Sciences, Mid-Sweden University, Östersund, SE- 831 25, Sweden

**Keywords:** Sedentary behaviour, Physical activity, Perceived physical environment, Educational level, Income, Gender

## Abstract

**Background:**

Sedentary behaviour in general and sitting time in particular is an emerging global health concern. The aim of this study was to provide data on the prevalence of sitting time in German adults and to examine socio-demographic and environmental correlates of sitting time.

**Methods:**

A representative sample of German adults (n = 2000; 967 men, 1033 women; 49.3 ±17.6 years of age) filled in the Global Physical Activity Questionnaire, including one question on overall sitting time and answered questions about the neighbourhood environment, as well as concerning demographics. Daily sitting time was stratified by gender, age group, BMI, educational and income level, as well as physical activity (PA). To identify socio-demographic and environmental correlates of sitting time, we used a series of linear regressions.

**Results:**

The overall median was 5 hours (299 minutes) of sitting time/day and men sat longer than women (5 vs. 4 hours/day; p < 0.05). In both genders age and PA were negatively and the educational level positively associated with sitting time. The level of income was not a correlate of sitting time in multivariate analyses. Sitting time was significantly positively associated with higher neighbourhood safety for women. The variance of the multivariate model ranged from 16.5% for men to 8.9% for women.

**Conclusions:**

The overall sitting time was unequally distributed in the German adult population. Our findings suggest implementing specific interventions to reduce sitting time for subgroups such as men, younger aged adults and adults with a higher education and lower PA. Future studies should enhance our understanding of the specific correlates of different types and domains of sitting in order to guide the development of effective public health strategies.

## Background

Sedentary behaviour in general and sitting time in particular are highly prevalent in all population groups and reflect a social and physical environment that supports sitting during daily life [1,2]. The common assumption that sufficient moderate-to-vigorous physical activity (PA) can compensate for sedentary behaviour has to be corrected since such behaviour has been found to increase the risk of various negative health outcomes independently of PA levels [3]. Evidence shows that sedentary behaviour is consistently associated with an increased risk of all-cause mortality [3,4] and is associated with various other negative health conditions such as obesity [5-7], cardiovascular diseases [8,9], type 2 diabetes mellitus [10], as well as various other metabolic risk factors [6,11]. Thus, sedentary behaviour is an emerging global health concern. Correlates of sedentary behaviour need to be understood and populations at risk identified to better address future public health action.

There is still, however, some confusion concerning the distinction between being inactive and being sedentary. Sedentary behaviour is defined by any waking behaviours that result in low energy expenditure in the range of 1.0–1.5 METs (< 1.5 times the resting energy expenditure) [12] and includes activities such as lying down, sitting, watching television or using the computer. Thus, sitting has been highlighted as a specific marker of sedentary behaviours [13]. The Sedentary Behaviour Research Network (2012) recommends defining inactivity in contrast to being sedentary as not meeting health-related PA recommendations [12].

In the last few decades most attention has been paid to monitoring and understanding the correlates of PA [14], as well as promoting sufficient PA [15,16]. Although data on sedentary behaviour is evolving [2,17,18], more information is needed to understand the distribution and correlates of sitting in different population groups. The heterogeneity of overall sitting time between countries of different continents has been documented, with reports indicating the lowest median values in Portugal, Brazil and Colombia (medians ≤ 180 min/day) and the highest values in Taiwan, Norway, Hong Kong, Saudi Arabia and Japan (medians ≥ 360 min/day). Great differences have been found even between European countries [17]. German data on sitting time were last collected in 2002 [19] and showed that 43.4% of the sample were sitting more than 6 hours a day, as a proxy for prolonged sitting time. Detailed analyses of sitting time for German adults stratified socio-demographically are lacking.

Studies examining correlates of sedentary behaviour are in an early stage and, in most cases, limited to TV viewing. Further research on these socio-demographic findings is warranted to better understand the phenomenon as such and to identify relevant target groups in order to develop effective interventions. Furthermore, the findings concerning the association between PA and sitting time are inconsistent [17,20,21]. Some of the recently published studies show no association between PA and sitting time [21], whereas others show a negative association [17], and still others investigating different domains of sedentary behaviour suggest gender-specific associations [20,22].

In terms of an ecological approach, it is important to understand environmental correlates [23]. Different studies observed some relations of sedentary behaviour with the physical environment. Living in high walkable neighborhoods compared to low walkable neighbourhoods was correlated with TV viewing time for women in Australia [24] and with vehicle miles travelled in automobiles in the US [25]. Contradictory findings concerning the association between walkability and overall sitting time (self-reported and accelerometer-based) were reported in Belgium [26]. Pooled analyses from environmentally diverse countries (USA, Australia, and Belgium) showed that motorized transport was negatively and linearly associated with a specific index of self-reported attributes of the neighbourhood environment (e.g. walking and cycling facilities, number of destinations, traffic safety). Overall sitting time as a more generic measure of sedentary behaviour was less consistently associated with an index of the environmental attributes land use mix-diversity, proximity of destinations and aesthetics [27].

As sedentary behaviour, including prolonged sitting is an independent risk factor for a variety of health concerns, it is important to specifically inform on the prevalence of sitting time and examine potential correlates of sitting as a prerequisite for the development of interventions [23]. Consequently, the aim of this study was 1) to provide data on the prevalence of sitting time in Germany, and 2) to investigate possible associations between sitting time and socio-demographic variables (gender, age, BMI, income groups, education level) and PA level as well as neighbourhood environmental variables.

## Methods

### Study design

A nationwide study on self-reported health behaviours was conducted in Germany. The sample size was set to 2000 citizens who were representative for the distribution of the German population. The service research centre ‘Growths from Knowledge’ (GfK) in Nuremberg collected the data between March and April 2010 as part of a computer-assisted telephone interview (CATI). The questions about self-reported sitting time and PA were nested into the population survey on health behaviour. The selected professional interviewers were trained in administering the computer-assisted standardized questionnaire. All study procedures were approved by the Ethics Committee of the German Sport University in Cologne.

### Study population

Two thousand representative residents (967 men, 1033 women) in the 16 German federal states over 18 years of age (mean 49.3 ± 17.6) were interviewed. The sample was taken from the ‘ADM pool for telephone samples’ (ADM = **A**rbeitskreis der **d**eutschen **M**arkt- und Sozialforschungsinstitute – a study group of German market and social research institutions). The ADM pool is a precisely co-ordinated national sample based on all possible telephone numbers, which forms the basis for selecting a population sample in the Federal Republic of Germany. The sample was weighted to the German population (year 2010) by age, gender, federal state, residential density and household size according to the data from the National Federal Statistical Office. The response rate to reach the sample size of 2000 respondents was 9.2%, probably mainly caused by the overall length of the survey of more than 25 minutes that may result in a high drop-out rate. Considering the methodology-related literature on surveys [28,29], the present response rate still seems acceptable for investigating the stated research question.

### Measures

#### Sitting time and physical activity

The Global Physical Activity Questionnaire (GPAQ) was used to assess sitting time and PA [30]. A single question in the GPAQ asked about sitting time: ‘How much time do you usually spend sitting or reclining on a typical day?’ The interviewers explained that the question was about sitting or reclining at work, at home, when getting to and from places, or with friends, including time spent sitting at a desk, sitting with friends, travelling in a car, bus or train, reading, playing cards or watching television, but did not include time spent sleeping [30]. The question was answered in terms of hours and minutes. The average sitting time per day in minutes was calculated as a continuous variable, as well as a dichotomous variable referring to a cut-off of 6 hours sitting. The latter variable was calculated to be comparable with recent German data [19].

PA was assessed in three domains: work (paid and unpaid work, including household chores), transport and leisure [30]. In the work and leisure domains, information on the frequency and duration of vigorous- as well as moderate-intensity PA were obtained. For the transport domain, information on all walking and cycling activities was included without differentiation of the intensity. Weekly minutes of moderate- and vigorous-intensity activity were calculated separately by multiplying the number of days per week by the duration on an average day. Reported minutes per week in each category were multiplied by the metabolic energy turnover (MET) equivalent, which is generally used to express the intensity of PA regardless of body weight. Four METs corresponded to the time spent in moderate-intensity activities and eight METs corresponded to the time spent in vigorous-intensity activities [31]. PA levels were classified into ‘low’, ‘moderate’ or ‘high’ according to the definition given by the GPAQ analysis framework [31].

All GPAQ data were checked for possible data entry errors by using the ‘CleanRecode’ program ( http://www.who.int/chp/steps/resources/database/en/index.html) provided by WHO.

The validity and reliability of the GPAQ has been assessed. The concurrent validity between the International Physical Activity Questionnaire (IPAQ) and the GPAQ showed a moderate to strong positive relationship (range 0.45 to 0.65) and reliability was of moderate to substantial strength (kappa 0.67 to 0.73; Spearman's rho 0.67 to 0.81) [32]. The concurrent validity of the sitting question was good (r = 0.65). The pooled criterion validity from pedometer studies for time spent in sedentary activities produced a fair negative correlation (r=0.26) [30] and self-reported sitting time has been found to be significantly and positively correlated with the time spent in sedentary behaviour assessed by accelerometers [30,33].

#### Socio-demographic variables

Demographic variables measured self-reported age, gender and body mass index (calculated using self-reported body weight and body height according to the formula BMI = m/kg^2^). Further socio-demographic variables included the educational and income level. The educational level was categorized into the following levels based on the German school system: no school graduation, 10 years of education, 12 years of education, 13 years of education and first university degree or higher. Household net income per month was assessed in nine categories and summarized in 3 groups: low income (< 1500€), middle income (1500€–3499€), and high income (€>3500€).

#### Environmental variables

The assessment of the perceived environment was self-administered using a modified version of the German short form of the European Environmental Questionnaire ALPHA [34], which includes ten items. For the analyses, we included only seven variables considering the neighbourhood environment and excluded three variables looking at the home and work environment. Instead of the dichotomized response scaling (yes vs. no) in the original version, we used a five-point rating scale (strongly disagree to strongly agree) to maintain the main response scaling in the whole survey. The questions covered six themes of the neighbourhood environment: types of residences (1 item), distances to local facilities (1 item), public transport infrastructure (1 item), access to parks and recreation facilities (1 item), neighbourhood safety (2 items) and pleasure, as well as ‘aesthetics’ of the neighbourhood (1 item). All items with a higher score, indicating a less supportive environment for PA, were recoded so that a higher score referred to a more supportive environment for PA. The original instrument was translated from English into German, followed by cognitive testing [34]. The performance of the modified instrument is unknown, whereas the ICC of the total sum score of the original ALPHA short was 0.73, which indicates good test-retest stability [34].

### Statistical analysis

All analyses were conducted using PASW Statistics 20 for Windows. Means, standard deviations and medians were calculated for sitting time. In addition, data on reported sitting time were categorized into the prevalence of ‘prolonged sitting’ (> 6 hours per day). The sample distribution in the variable ‘sitting time’ was slightly skewed (SK = 0.86). Different transformations [35] did not improve the normality of the distribution. Therefore, we decided not to transform the variable. ANOVA analyses were performed to examine differences between subgroups. Multiple linear regression analyses were executed to investigate associations of socio-demographic, behavioural and environmental correlates and the dependent variable sitting time for men and women separately. Referring to an ecological approach to sedentary behaviour [23], we chose the forced entry method to explore the associations with sitting time. Socio-demographic variables included age (continuous variable), BMI (continuous variable), education (four categories) and income level (three categories). The behavioural variables consisted of total PA MET minutes per week (continuous variable) and the seven environmental variables (each five-point scale). All variables included in the model were assessed for multicollinearity. We did not observe a correlation coefficient above 0.4 or a variance inflation factor greater than 2 between all pairs of the independent variables [35]. Statistical significance was set at a level of 0.05.

## Results

Table 1 shows the frequency distribution of the population sample stratified by gender and gives the national representative figures for the German population [36]. The representativeness of the study population is given for age, gender and income level. The educational level seems to be higher compared to the overall German population.

**Table 1:** Sample characteristics stratified by gender (n = 2000) (n.a. = not available)

	**Sample population**			German population in 2010*		
	**All**	**Men**	**Women**	**All**	**Men**	**Women**
	**(n = 2000)**	**(n = 967)**	**(n = 1033)**			
	**n (%)**	**n (%)**	**n (%)**	**%**	**%**	**%**
**Sex**						
Male	967					
	(48.8)			48.6		
Female	1033					
	(51.6)			51.4		
Age*						
18–29 years	335 (16.7)	190 (19.7)	145 (14.0)	17.2	18.1	16.5
30–45 years	550 (27.5)	250 (25.8)	300 (29.1)	24.7	25.8	23.6
46–65 years	644 (32.2)	302 (31.2)	342 (33.1)	33.4	34.4	32.4
≥ 66 years	471 (23.6)	226 (23.3)	245 (23.8)	24.7	21.7	27.6
**BMI**						
<18.5 kg/m^2^	36 (1.8)	16 (1.7)	20 (2.0)	n.a.	n.a.	n.a.
18.5–24.99 kg/m^2^	1050 (53.9)	454 (47.7)	596 (59.8)	n.a.	n.a.	n.a.
>25 kg/m^2^	862 (43.1)	481 (50.6)	380 (38.2)	n.a.	n.a.	n.a.
**Income groups** household net income/month	*(n = 1764)*	*(n = 868)*	*(n = 896)*			
<1500€	685 (38.8)	278 (32.0)	407 (45.4)	36.6	n.a.	n.a.
1500-3499€	936 (53.1)	502 (57.8)	434 (48.5)	55.4**	n.a.	n.a.
>3.500€	143 (8.1)	89 (10.2)	54 (6.1)	7.9***	n.a.	n.a.
**Educational level**	*(n = 1973)*	*( n = 957)*	*(n = 1016)*			
No graduation	22 (1.1)	14 (1.4)	8 (0.8)	n.a.	n.a.	n.a.
10 years	350 (17.8)	153 (16.0)	197 (19.4)	39.3****	n.a.	n.a.
12 years	689 (34.9)	312 (32.6)	377 (37.1)	21.1	n.a.	n.a.
13 years	520 (26.4)	250 (26.2)	270 (26.5)	24.4	n.a.	n.a.
University degree	392 (19.9)	228 (23.8)	164 (16.2)	13.6	n.a.	n.a.

* [36].

** German population proportion of national household net income per month in 2010 for the range of 1.500-4.500€.

*** German population proportion of national household net income per month in 2010 for > 4.500€.

**** German population proportion of educational attainment of the population in Germany in 2010 with ≤ 10 years education (general secondary school-leaving certificate).

Overall, the median reported sitting time was just under 5 hours per day (299 minutes/day) with an average of 317 ± 185 minutes/day in the German population. The median for men was 1 hour/day higher than for women (5 hours/day [300 min/day] vs. 4 hours/day [240 min/day]; p < .05), while the ≥ 66-year-old men sat the shortest period of time (p < .05). Among women, 18–29-year-olds sat longer than the older age groups (p < .05). For men, sitting time in the lowest income group was lower than in the highest income group (p <. 05) and participants with an educational duration of 13 years or more had longer sitting times than participants with 10 and 12 years of education (p < .05). Men and women with higher PA levels reported less sitting time than participants with low or moderate PA levels (p <. 05) (see Table 2).

**Table 2:** Mean and standard deviation (median) for sitting time in minutes/day and prevalence of prolonged sitting of 6 hours and more for age, BMI, income groups, educational and PA levels, stratified by gender (n = 1986)

	**All (n = 1986)**		**Men (n = 961)**		**Women (n = 1024)**	
	x ± s (median)	> 6 hours (%)	x ± s (median)	> 6 hours (%)	x ± s (median)	> 6 hours (%)
	316.7 ± 184.8 (299)	30.1	340.5 ± 191.3 (300)^a^	36.1	294.3 ± 175.7 (240)	24.5
**Age**						
18–29 years	369.5 ± 190.5 (360)^b,c,d^	45.4	375.5 ± 193.8 (360)^d^	48.6	361.6 ± 186.5 (360)^b,c,d^	41.2
30–45 years	329.8 ± 199.1 (300)^e^	35.0	375.4 ± 205.9 (360)^e^	45.5	292.1 ± 185.3 (240)	26.4
46–65 years	316.4 ± 180.8 (296.7)^f^	30.4	345.5 ± 191.0 (300^)f^	37.0	290.4 ± 167.2 (240)	24.4
≥66 years	263.9 ± 153.0 (240)	13.2	264.9 ± 147.4 (240)	13.8	262.9 ± 158.2 (240)	12.7
**BMI**						
< 18.5 kg/m^2^	332.9 ± 220.4 (360)	34.9	416.0 ± 219.3 (425)	55.5	269.9 ± 204.4 (278)	18.3
18.5–24.99 kg/m^2^	317.4 ± 182.1 (299)	31.0	342.1 ± 192.3 (300)	37.3	298.6 ± 171.8 (240)	26.3
> 25 kg/m^2^	312.6 ± 183.6 (270)	27.6	335.2 ± 188.2 (300)	33.5	283.7 ± 173.6 (240)	20.2
**Income groups** (household net income/month)						
< 1500 €	300.1 ± 180.2 (240)^g^	26.7	313.1 ± 182.5 (251.4)^g^	30.4	291.2 ± 178.3 (240)	24.2
1500–3499€	317.1 ± 183.6 (299)^h^	29.2	341.2 ± 190.9 (300)	35.3	288.1 ± 170.1 (240)	22.0
> 3.500€	358.4 ± 185.8 (360)	41.7	381.5 ± 188.5 (360)	48.0	329.7 ± 179.2 (300)	33.8
**Educational level**						
No graduation	311.4 ± 161.2 (270.7)	35.7	291.7 ± 149.5 (239.1)	22.4	342.1 ± 184.0 (419)	56.3
10 years	253.9 ± 164.1 (240)^i,j,k^	14.8	265.3 ± 170.2 (240)^i,j,k^	19.4	245.1 ± 159.2 (240)^j,k^	11.2
12 years	300.8 ± 177.6 (240)^l,m^	27.2	322.5 ± 183.9 (299.9)^l^	32.1	282.8 ± 170.3 (240)^l,m^	23.0
13 years	355.4 ± 191.4 (355.9)	39.3	388.3 ± 198.9 (360)	47.9	324.5 ± 179.1(300)	31.3
University degree	352.2 ± 186.9 (330)	37.8	368.5 ± 191.8 (360)	41.8	329.6 ± 178.1 (300)	32.2
**PA level**						
Low	399.9 ± 219.8 (360)^n^	49.4	431.1 ± 218.2 (480)^n^	56.1	370.9 ± 217.8 (359)^n^	43.3
Moderate	332.9 ± 183.9 (300)^o^	33.1	360.4 ± 191.1 (360)^o^	40.1	306.8 ± 173.6 (270)^o^	26.5
High	285.6 ± 164.6 (240)	23.3	306.1 ± 172.4 (270)	28.7	266.3 ± 154.7 (240)	18.2

^a^ Men differ significantly from women (p < 0.05).

^b^ Age group 18–29 years differs significantly from age group 30–45 years (p < 0.05).

^c^ Age group 18–29 years differs significantly from age group 46–65 years (p < 0.05).

^d^ Age group 18–29 years differs significantly from age group > 66 years (p < 0.05).

^e^ Age group 30–45 years differs significantly from age group > 66 (p < 0.05).

^f^ Age group 46–65 years differs significantly from age group > 66 (p < 0.05).

^g^ Subjects in the lowest income group differ significantly from subjects in the highest income group (p < 0.05).

^h^ Subjects in the middle income group differ significantly from subjects in the highest income group (p < 0.05).

^i^ Subjects with an education of 10 years differ significantly from subjects with an education of 12 years (p < 0.05).

^j^ Subjects with an education of 10 years differ significantly from subjects with an education of 13 years (p < 0.05).

^k^ Subjects with an education of 10 years differ significantly from subjects with a first university degree or higher (p < 0.05).

^l^ Subjects with an education of 12 years differ significantly from subjects with an education of 13 years (p < 0.05).

^m^ Subjects with an education of 12 years differ significantly from subjects with an with a first university degree or higher (p < 0.05).

^n^ Subjects in the low PA group differ significantly from subjects in the moderate PA group (p < 0.05).

^o^ Subjects in the moderate PA group differ significantly from subjects in the high PA group (p < 0.05).

In Table 2 the prevalence of sitting for 6 hours or more per day is also shown. For the total sample, the prevalence of prolonged sitting was 30.1%. The highest prevalence was found for 18–29-year-old men (48.6%), men with a monthly household net income of > 3.500€ (48.0%), men with 13 years of education (47.9%) and men with a low PA level (56.1%). The lowest prevalence of prolonged sitting was reported among participants aged 66 years and older (13.2%), women (12.7%) and women with an educational duration of 10 years (11.2%).

Multiple linear regressions were computed for men (n = 830) and women (n = 834) separately (see Table 3). Multivariate regression analyses showed that 16.5% of the variance (adjusted R^2^) in men and 8.9% of the variance in women were explained by the variables entered in the model. Age and PA were negatively associated with sitting time, indicating that increasing age and PA led to a reduction in sitting time in both genders. For men and women, ‘education’ was positively associated with ‘sitting time’, meaning that an increasing educational level was correlated with increasing sitting time. Only for women was the environmental variable ‘Walking is unsafe because of the traffic in my neighbourhood’ (β = .07) positively correlated with sitting time suggesting increasing sitting duration with higher neighborhood safety.

**Table 3:** Results from multiple linear regressions on the contribution of multidimensional correlates on the dependant variable “sitting time” for males (n = 830) and females (n = 834) (B = unstandardized beta; SE B = standard error of beta; β = standardized beta; * = p < 0.05; ** = p < 0.01; *** = p < 0.001)

	**Males (n = 830)**			**Females (n=834)**		
	**B**	**SE B**	**β**	**B**	**SE B**	**β**
Age	−2.40	0.36	-.23***	−1.30	0.37	-.13***
BMI	2.78	1.46	.06	0.75	1.24	.02
Educational level	21.38	6.15	.12**	21.65	6.29	.13**
Income level	18.82	9.89	.07	2.89	9.04	.01
PA level	−0.04	0.00	-.27***	−0.03	0.00	-.21***
Most of the houses in my neighbourhood are detached houses^a^	7.37	3.83	.07	5.85	3.68	.06
Many shops, stores, markets or other places to buy things I need are within easy walking distance of my home^b^	−2.48	4.29	-.02	−2.19	3.87	-.02
There is a transit stop (such as a bus stop, train, trolley or tram station) within easy walking distance of my home^b^	−1.19	6.72	-.01	4.48	5.73	.03
There is an open recreation area (e.g. park, beach or other open space) within easy walking distance of my home^b^	3.96	5.92	.02	−5.90	5.46	-.04
Walking is unsafe because of the traffic in my neighbourhood^a^	9.10	5.63	.06	9.73	4.76	.08*
Walking is unsafe because of the level of crime in my neighbuorhood^a^	11.64	5.97	.07	−6.96	5.14	-.05
In my neighbourhood there are trees along the streets^b^	−0.18	4.58	-.001	−2.52	4.54	-.02

*Adj. R*^*2*^= .165 for males; *Adj. R*^*2*^= .089 for females.

^a^ Response option: strongly agree (1), somewhat agree (2), in between (3) disagree somewhat (4), strongly disagree (5).

^b^ Response options were recoded into: strongly disagree (1), disagree somewhat (2), in between (3), somewhat agree (4), strongly agree (5).

## Discussion

The results showed a generally high level of overall sitting time of 5 hours/day in the German population, with men sitting significantly longer than women. In both genders age and PA were negatively associated and the educational level was positively associated with sitting time. Interestingly, the level of income did not significantly contribute as an independent correlate of sitting time. Only one environmental correlate ‘Walking is unsafe because of the traffic in my neighbourhood’ was independently associated with sitting time in women. In men, no associations with environmental correlates were found. The overall variance of the multivariate model ranged from 16.5% for men to 8.9% for women.

### Prevalence

The median sitting time in the German population was 5 hours per day, which represents approximately 31% of an adult´s assumed 16 waking hours a day. Regarding the 20-country comparison [17], the results were congruent with the overall median of the investigated countries and similar to those in such investigated European countries as Belgium, Sweden or Spain. Compared to collected IPAQ data from the Netherlands, the UK and the USA, which showed sitting times ranging from 5.5 hours to >7 hours [21], the sitting time for the German population falls within the lower range. A possible explanation could be the use of a convenience sample in the study by Rosenberg et al. [21]. Their study consisted mainly of university staff and students with a generally high educational and socioeconomic status who may have overall higher sitting times, as also seen in the present study.

Regarding prolonged sitting times of six hours and more, the present study revealed a reduction in prevalence points of about 13.3 compared to the study sample in 2002 (30.1% vs. 43.4%) [19]. The extent of this finding was not expected and is of crucial importance to explain it. A possible explanation could be that the study samples are not entirely comparable due to a higher mean age and slightly higher income levels in the present study. Furthermore, the low response rate in the current study has to be considered as it implies a possible selection bias of health-interested respondents who answered the survey and reported less sitting time. In addition, it should be kept in mind that the cut-off level of > 6 hours is an artificial threshold and small shifts of minutes per day for people close to the cut-off might result in large differences.

Studies using objective measurements such as accelerometers to assess sitting time detected even higher durations for sedentary behaviour, for example in the United States with 7.7 hours/day [2], in China with 8,5 hours/day [37], or as in Australia where participants spend 57% of their waking hours sedentary [6]. It is well known that objective measurements have been associated with higher sedentary behaviour than self-reported behaviour. This reflects a higher sensitivity of objective measurements of overall sitting time and overcomes issues of recall bias of self-reported measures [38]. It is not possible to compare these numbers with German populations since there is lack of representative objectively collected data. However, the sitting item of the IPAQ, which in contrast to the GPAQ distinguishes between sitting time during weekdays and weekend day, but otherwise offers the same question phrasing, mirrored reasonable agreement compared to accelerometer counts/min <100 [21]. Nevertheless, studies using objective measurements to determine sitting time are warranted.

The significantly higher amount of sitting time among men in our study corresponds with that of past studies [2,39]. Bauman et al. [17] reported higher sitting times among men in seven out of 20 countries. Contradictory findings were reported from the US [38], indicating a lower prevalence of women in screen time, but a higher prevalence of women for ‘sitting most of the day’ than for men, resulting in a longer duration of overall sitting time for women with reference to accelerometer counts. Results from Australia pointed out that there were gender-specific dissimilarities on looking at the different domains of sitting for watching TV, general leisure and home computer use during the usual weekday and weekends [40]. To understand these gender-specific patterns of sitting time, it is necessary to examine in more detail, i.e. screen time, non-screen time or the different domains of sitting, such as at work, in transport and during leisure to develop well-directed interventions.

### Correlates of sitting time

The second aim of the present study was to explore sitting behaviour in respect to different socio-demographic and environmental correlates. Multivariate models examining the association between overall sitting time and the above-mentioned correlates explained more of the variance in men (R^2^ = 16.5%) than in women (R^2^ = 8.9%). From a public health perspective, the low variance might still be of significance for developing interventions of the population level to reduce sitting time. However, the results also showed that a large part of the model variance remains unexplained by the included correlates. Models including different correlates, such as social norms, psychosocial or home environment correlates (e.g. home entertainment, labour-saving devices) might be most promising for explaining sitting behaviour [23]. Consequently, on-going research has been assigned to investigate possible correlates of sitting, considering the different types and domains of sedentary behaviour [40] and recognizing the relevant contextual factors [23].

The present results confirmed decreasing sitting time with increasing age for both genders and replicated recent findings [17]. The greater use of technology, sitting occupations and passive modes of transport among younger adults could account for this behaviour. However, opposite age relationship patterns were found in other studies using self-reports [38], as well as objective measuring tools [2,37]. Reasons for this discrepancy could be seen in the more challenging task of answering the self-report sitting time question for older people. This might affect the accuracy of the response [41]. Furthermore, Healy et al. [38] indicated a sitting domain specific age-related influence, showing increasing sitting times with age for TV viewing and screen time, but decreasing values for computer use. Therefore, ongoing research that investigates the effect of age on sitting with objective as well as domain-specific self-report data management is warranted to better identify sitting patterns related to age.

The present finding that there is no association between overweight and sitting time can partly be explained by the results of a recent systematic review [3], which revealed only limited evidence for a longitudinal relationship between sedentary behaviour, weight gain, and the risk of obesity. Moreover, studies suggested a relationship between overweight and more specific aspects of sitting, such as TV watching [42], but not overall sitting time as collected in the present study. Also, self-reported BMI as in the present study may lead to misclassifications, which could explain the missing association.

Studies have shown that the level of education was positively associated with sitting time [17], especially during weekdays [43]. This was confirmed by our results for men and women and indicates that reasonable interventions to reduce sitting time have to be developed, especially for people with higher levels of education. However, studies investigating more specific sitting behaviours indicate that people with lower education have longer TV viewing time during leisure [40]. Interestingly, the income level was not independently associated with sitting time and fades in the model, which might be due to the fact that the correlate of income level ‘hides’ behind the educational level. Burton et al. [40] also did not reveal an overall association of sitting time with income level, but demonstrated longer home computer-use times in the mid-income group.

Based on our current findings of the socio-demographic correlates, we can conclude that the main target groups for reducing overall sitting time are especially men and younger and more educated adults. This might be a surprising conclusion as it is different from what we know from the field of PA promotion. Future studies should focus on contextual factors considering the domain and the type of sedentary behaviour to develop effective action for high-risk groups such as men or perhaps managers, university students, office workers etc. in order to reduce sitting time. However, considering measurement issues (e.g. response bias increasing with age) and the versatile nature of sedentary behaviour as a distinct class of behaviours, future studies must identify target groups depending on their dominant sedentary behaviour instead of overall sitting time.

The present findings suggest a strong negative association between PA and sitting time for both genders. Decreasing levels of PA have been associated with increasing overall sitting time before [5,17,19]. However, it has to be emphasized that the evidence is not consistent in this matter and several studies detected no association [21], inconsistent association [44] or even positive associations, indicating that PA and sitting behaviour are independent constructs [20]. Keeping in mind that in the present study all domains of PA (work including household chores, transport and leisure) as well as overall sitting time were assessed it also seems reasonable that people with high PA do not report on high sitting time, because of the limited time. Especially studies looking at distinct sitting behaviours during leisure time and specific leisure PA did not find negative associations between PA and sitting [20]. Consequently, domain-specific studies, looking at PA as well as sitting behaviour, are required.

Overall, the association between the environmental correlates and overall sitting time was weak in the present study, which may be due to the fact that the environmental questions, which were based on the ALPHA questionnaire, were developed for a PA context and not for sitting. However, we found a significant association for women between a higher perceived neighborhood safety and an increasing overall sitting duration. This finding was unexpected and may originate from a selection bias in that people with higher educational and income levels choose safer neighborhoods which was associated with longer sitting times. A further explanation could be the missing distinction of sedentary behaviour domains (household, leisure time, transport and occupation) as suggested by the ecological model [23]. This may also be one rationale for the missing association between overall sitting time and the other environmental correlates in the present study. Here, investigations of the association between more specific sitting times, i.e. time during motorized transport and environmental correlates, could be promising [27]. All in all, it has to be emphasized that research considering a possible association between sitting time and environmental correlates is just evolving and that future studies need to investigate the specificity of the environment (home, neighborhood, recreation and workplace environments) and the diverse domains of sitting, for example, investigating the neighbourhood environmental correlates of time sitting in cars or home environmental correlates of leisure-time sitting and screen-based entertainment sitting time [23].

### Limitations and strengths

Although the sample was representative of the German population concerning age, gender, federal state, residential density and household size, the low response rate in the study is a limitation. Nevertheless, referring to the overall decline of response rates during recent decades [28] and considering survey research showing that no difference in empirical findings was a given characteristic of study protocols which accepted a low response rate as compared to studies with a higher response rate due to more aggressive attempts to make contact [29], the present response rate seems acceptable and appropriate for investigating the given research question. However, the potential for a survey non-response bias or a selection bias of the health-interested population should be acknowledged. A further limitation in this study is the outcome of ‘overall sitting time’, with no differentiation between weekdays and weekend days and no domain-specific information concerning sitting behaviour. Furthermore, our information on sitting time was obtained by self-report. Consequently, our results might be biased due to misclassifications or social desirability. Future research should use both objective and subjective assessments of sitting time to capture important domain- and behaviour-specifıc sitting time information on weekdays and weekend days and to objectively measure total sitting time, as well as patterns of sitting [38]. Another limitation in this study is the adaption of the response scale from the ALPHA questionnaire, which may have an impact on the validity of the questions and may aggravate comparability with other research including environmental correlates. Strengths of this study include the reasonably large sample size and the inclusion of correlates of multiple domains in terms of understanding health behaviours.

## Conclusion

The present study gives first insights into overall sitting time and possible correlates for the German adult population. Prolonged sitting is an emerging public health problem which needs to be prevented in order to avoid its negative health consequences. Further research is warranted to investigate domain-specific sitting time and identify subgroups that have specific needs in order to guide policy-makers in developing promising interventions to reduce sitting time. Only weak associations with environmental correlates were seen. Here, future research needs to address the specificity of the environment and possible associations with specific domains of sitting to obtain more fundamental insights into these associations.

## Abbreviations

PA: Physical activity; GPAQ: Global Physical Activity Questionnaire; IPAQ: International Physical Activity Questionnaire

## Competing interests

The authors declare that they have no financial or non-financial competing interests.

## Authors’ contributions

BW participated in the conception and the design of the present study and performed statistical analyses interpreted the data and wrote and drafted the manuscript. SH gave support in literature research, data calculations and writing the manuscript. JB and PS contributed to the analyses and interpretation of data and provided critical revision of the manuscript. IF participated in the conception and design of the study. All authors read and approved the final manuscript.

## Pre-publication history

The pre-publication history for this paper can be accessed here:

http://www.biomedcentral.com/1471-2458/13/196/prepub

## References

[B1] Owen N Healy GN Matthews CE Dunstan DW Too much sitting: the population health science of sedentary behavior Exerc Sport Sci Rev 2010 38 3 105 113 10.1097/JES.0b013e3181e373a2 20577058 PMC3404815

[B2] Matthews CE Chen KY Freedson PS Buchowski MS Beech BM Pate RR Troiano RP Amount of time spent in sedentary behaviors in the United States, 2003–2004 Am J Epidemiol 2008 167 7 875 881 10.1093/aje/kwm390 18303006 PMC3527832

[B3] Thorp AA Owen N Neuhaus M Dunstan DW Sedentary behaviors and subsequent health outcomes in adults a systematic review of longitudinal studies, 1996–2011 Am J Prev Med 2011 41 2 207 215 10.1016/j.amepre.2011.05.004 21767729

[B4] Dunstan DW Barr EL Healy GN Salmon J Shaw JE Balkau B Magliano DJ Cameron AJ Zimmet PZ Owen N Television viewing time and mortality: the Australian Diabetes, Obesity and Lifestyle Study (AusDiab) Circulation 2010 121 3 384 391 10.1161/CIRCULATIONAHA.109.894824 20065160

[B5] Martinez-Gonzalez MA Martinez JA Hu FB Gibney MJ Kearney J Physical inactivity, sedentary lifestyle and obesity in the European Union Int J Obes Relat Metab Disord 1999 23 11 1192 1201 10.1038/sj.ijo.0801049 10578210

[B6] Healy GN Wijndaele K Dunstan DW Shaw JE Salmon J Zimmet PZ Owen N Objectively measured sedentary time, physical activity, and metabolic risk: the Australian Diabetes, Obesity and Lifestyle Study (AusDiab) Diabetes Care 2008 31 2 369 371 18000181 10.2337/dc07-1795

[B7] Santos R Soares-Miranda L Vale S Moreira C Marques AI Mota J Sitting time and body mass index, in a Portuguese sample of men: results from the Azorean Physical Activity and Health Study (APAHS) Int J Environ Res Public Health 2010 7 4 1500 1507 10.3390/ijerph7041500 20617042 PMC2872332

[B8] Warren TY Barry V Hooker SP Sui X Church TS Blair SN Sedentary behaviors increase risk of cardiovascular disease mortality in men Med Sci Sports Exerc 2010 42 5 879 885 10.1249/MSS.0b013e3181c3aa7e 19996993 PMC2857522

[B9] Dunstan DW Thorp AA Healy GN Prolonged sitting: is it a distinct coronary heart disease risk factor? Curr Opin Cardiol 2011 26 5 412 419 10.1097/HCO.0b013e3283496605 21785350

[B10] Ford ES Schulze MB Kroger J Pischon T Bergmann MM Boeing H Television watching and incident diabetes: findings from the European Prospective Investigation into Cancer and Nutrition-Potsdam Study J Diabetes 2010 2 1 23 27 10.1111/j.1753-0407.2009.00047.x 20923471

[B11] Sisson SB Camhi SM Church TS Martin CK Tudor-Locke C Bouchard C Earnest CP Smith SR Newton RL Jr Rankinen T Leisure time sedentary behavior, occupational/domestic physical activity, and metabolic syndrome in U.S. men and women Metab Syndr Relat Disord 2009 7 6 529 536 10.1089/met.2009.0023 19900152 PMC2796695

[B12] Sedentary Behaviour Research N Letter to the editor: standardized use of the terms "sedentary" and "sedentary behaviours" Appl Physiol Nutr Metab 2012 37 3 540 545 10.1139/h2012-024 22540258

[B13] Owen N Bauman A Brown W Too much sitting: a novel and important predictor of chronic disease risk? Br J Sports Med 2009 43 2 81 83 19050003 10.1136/bjsm.2008.055269

[B14] Bauman AE Reis RS Sallis JF Wells JC Loos RJF Martin BW Correlates of physical activity: why are some people physically active and others not? Lancet 2012 380 9838 258 271 10.1016/S0140-6736(12)60735-1 22818938

[B15] Haskell WL Lee IM Pate RR Powell KE Blair SN Franklin BA Macera CA Heath GW Thompson PD Bauman A Physical activity and public health: updated recommendation for adults from the American College of Sports Medicine and the American Heart Association Circulation 2007 116 9 1081 1093 17671237 10.1161/CIRCULATIONAHA.107.185649

[B16] Bauman A Bull F Chey T Craig CL Ainsworth BE Sallis JF Bowles HR Hagstromer M Sjostrom M Pratt M The International Prevalence Study on Physical Activity: results from 20 countries Int J Behav Nutr Phys Act 2009 6 1 21 10.1186/1479-5868-6-21 19335883 PMC2674408

[B17] Bauman A Ainsworth BE Sallis JF Hagstromer M Craig CL Bull FC Pratt M Venugopal K Chau J Sjostrom M The Descriptive Epidemiology of Sitting A 20-Country Comparison Using the International Physical Activity Questionnaire (IPAQ) Am J Prev Med 2011 41 2 228 235 10.1016/j.amepre.2011.05.003 21767731

[B18] Hagstromer M Troiano RP Sjostrom M Berrigan D Levels and patterns of objectively assessed physical activity–a comparison between Sweden and the United States Am J Epidemiol 2010 171 10 1055 1064 10.1093/aje/kwq069 20406758

[B19] Sjöström M Oja P Hagströmer M Smith B Bauman A Health-enhancing physical activity across European Union countries: the Eurobarometer study J Public Health 2006 14 5 291 300 10.1007/s10389-006-0031-y

[B20] Burton NW Khan A Brown WJ Turrell G The association between sedentary leisure and physical activity in middle-aged adults Br J Sports Med 2012 46 10 747 752 10.1136/bjsm.2010.081430 21536709

[B21] Rosenberg DE Bull FC Marshall AL Sallis JF Bauman AE Assessment of sedentary behavior with the International Physical Activity Questionnaire J Phys Act Health 2008 5 Suppl 1 S30 S44 18364524 10.1123/jpah.5.s1.s30

[B22] Sugiyama T Healy GN Dunstan DW Salmon J Owen N Is television viewing time a marker of a broader pattern of sedentary behavior? Ann Behav Med 2008 35 2 245 250 10.1007/s12160-008-9017-z 18357498

[B23] Owen N Sugiyama T Eakin EE Gardiner PA Tremblay MS Sallis JF Adults' sedentary behavior determinants and interventions Am J Prev Med 2011 41 2 189 196 10.1016/j.amepre.2011.05.013 21767727

[B24] Sugiyama T Salmon J Dunstan DW Bauman AE Owen N Neighborhood walkability and TV viewing time among Australian adults Am J Prev Med 2007 33 6 444 449 10.1016/j.amepre.2007.07.035 18022059

[B25] Frank LD Saelens BE Powell KE Chapman JE Stepping towards causation: do built environments or neighborhood and travel preferences explain physical activity, driving, and obesity? Soc Sci Med 2007 65 9 1898 1914 10.1016/j.socscimed.2007.05.053 17644231

[B26] Van Dyck D Cardon G Deforche B Sallis JF Owen N De Bourdeaudhuij I Neighborhood SES and walkability are related to physical activity behavior in Belgian adults Prev Med 2010 50 S74 S79 19751757 10.1016/j.ypmed.2009.07.027

[B27] Van Dyck D Cerin E Conway TL De Bourdeaudhuij I Owen N Kerr J Cardon G Frank LD Saelens BE Sallis JF Associations between perceived neighborhood environmental attributes and adults' sedentary behavior: Findings from the USA 2012 Soc Sci Med: Australia and Belgium 10.1016/j.socscimed.2012.01.018 PMC3321105 22405686

[B28] Curtin R Presser S Singer E Changes in Telephone Survey Nonresponse over the Past Quarter Century Public Opin Q 2005 69 1 87 98 10.1093/poq/nfi002

[B29] Davern M McAlpine D Beebe TJ Ziegenfuss J Rockwood T Call KT Are lower response rates hazardous to your health survey? An analysis of three state telephone health surveys Health Serv Res 2010 45 5 Pt 1 1324 1344 20579127 10.1111/j.1475-6773.2010.01128.x PMC2965507

[B30] Armstrong T Bull F Development of the World Health Organization Global Physical Activity Questionnaire (GPAQ) J Public Health 2006 14 2 66 70 10.1007/s10389-006-0024-x

[B31] Global Physical Activity Questionnaire (GPAQ) Analysis Guide www.who.int/entity/chp/steps/resources/GPAQ_Analysis_Guide.pdf

[B32] Bull FC Maslin TS Armstrong T Global physical activity questionnaire (GPAQ): nine country reliability and validity study J Phys Act Health 2009 6 6 790 804 20101923 10.1123/jpah.6.6.790

[B33] Ekelund U Sepp H Brage S Becker W Jakes R Hennings M Wareham NJ Criterion-related validity of the last 7-day, short form of the International Physical Activity Questionnaire in Swedish adults Public Health Nutr 2006 9 2 258 265 16571181 10.1079/phn2005840

[B34] Spittaels H Verloigne M Gidlow C Gloanec J Titze S Foster C Oppert JM Rutter H Oja P Sjostrom M Measuring physical activity-related environmental factors: reliability and predictive validity of the European environmental questionnaire ALPHA Int J Behav Nutr Phys Act 2010 7 1 48 10.1186/1479-5868-7-48 20504339 PMC2892430

[B35] Tabachnick BG Fidell LS Using multivariate statistics 2007 5 Boston: Pearson Education

[B36] Federal Statistical Office: Statistical Yearbook 2011 For the Federal Republic of Germany including »International tables 2011 Wiesbaden: Federal Statistical Office

[B37] Peters TM Moore SC Xiang YB Yang G Shu XO Ekelund U Ji BT Tan YT da Liu K Schatzkin A Accelerometer-measured physical activity in Chinese adults Am J Prev Med 2010 38 6 583 591 10.1016/j.amepre.2010.02.012 20494234 PMC2897243

[B38] Healy GN Clark BK Winkler EA Gardiner PA Brown WJ Matthews CE Measurement of adults' sedentary time in population-based studies Am J Prev Med 2011 41 2 216 227 10.1016/j.amepre.2011.05.005 21767730 PMC3179387

[B39] Ottevaere C Huybrechts I Benser J De Bourdeaudhuij I Cuenca-Garcia M Dallongeville J Zaccaria M Gottrand F Kersting M Rey-Lopez JP Clustering patterns of physical activity, sedentary and dietary behavior among European adolescents: the HELENA study BMC Publ Health 2011 11 328 10.1186/1471-2458-11-328 PMC3112135 21586158

[B40] Burton NW Haynes M van Uffelen JG Brown WJ Turrell G Mid-aged adults' sitting time in three contexts Am J Prev Med 2012 42 4 363 373 10.1016/j.amepre.2011.11.012 22424249

[B41] van Uffelen J Heesch K Hill R Brown W A qualitative study of older adults' responses to sitting-time questions: do we get the information we want? BMC Publ Health 2011 11 1 458 10.1186/1471-2458-11-458 PMC3141448 21658274

[B42] Williams DM Raynor HA Ciccolo JT A review of TV viewing and its association with health outcomes in adults Am J Lifestyle Med 2008 2 3 250 259 10.1177/1559827608314104

[B43] Proper KI Cerin E Brown WJ Owen N Sitting time and socio-economic differences in overweight and obesity Int J Obes (Lond) 2007 31 1 169 176 10.1038/sj.ijo.0803357 16652126

[B44] van Uffelen JG Watson MJ Dobson AJ Brown WJ Comparison of self-reported week-day and weekend-day sitting time and weekly time-use: results from the Australian Longitudinal Study on Women's Health Int J Behav Med 2011 18 3 221 228 10.1007/s12529-010-9105-x 20526827

